# Evaluating Dyslipidemia and Atherogenic Indices as Predictors of Coronary Artery Disease Risk: A Retrospective Cross-Sectional Study

**DOI:** 10.7759/cureus.71187

**Published:** 2024-10-10

**Authors:** Joyce S Jose, Karra Madhu Latha, Aparna V Bhongir, Sangeetha Sampath, Anand K Pyati

**Affiliations:** 1 Biochemistry, All India Institute of Medical Sciences, Bibinagar, Hyderabad, IND

**Keywords:** atherogenic coefficient, atherogenic indices, cardiovascular disease, coronary artery disease (cad), dyslipidemia, hypercholesterolemia, hypertriglyceridemia, lipid profile, non-hdl

## Abstract

Background: The frequency of coronary artery disease (CAD) has alarmingly increased in India accounting for the majority of all fatalities. The primary risk factor for CAD, acute myocardial infarction, and stroke is dyslipidemia. CAD risk can be ascertained by the lipid profile as well as atherogenic risk indices. Due to the current scenario of increased CAD prevalence and the established role of dyslipidemia as a risk factor for CAD, this study aimed at identifying the prevalent lipid abnormalities in a tertiary care hospital in Telangana and studying the predictive value of atherogenic indices for assessing CAD risk.

Methods: This is a retrospective cross-sectional study. Data pertaining to the subjects from January 2021 to December 2021 was retrieved from the hospital records.

Results: Serum triglycerides (TG) were in the higher range for 47 % (n= 235) of total subjects and isolated Low-density lipoprotein (LDL) was higher in 12.6% (n = 63). The overall burden of dyslipidemia was 72.20% (n=361) with 57.6 % (n=208) in males and 42.4% (n=153) in females). The most common dyslipidemia found was Familial Combined Hyperlipidemia in 42.8% (n=214) of individuals. The second most common pattern of dyslipidemia was Primary Familial Hypercholesterolemia which was observed in 25% (n=125) of subjects. Dysbetalipoproteinemia was seen in 24.2% (n=121) of the study subjects. ROC analysis found the Atherogenic Coefficient (AC) to be the most sensitive and specific cardiovascular risk index. 57.8% of the subjects had AC >2.44 and were at the highest risk for developing CAD.

Conclusions: The overall burden of dyslipidemia was 72.2%. AC was found to be the most sensitive and specific cardiovascular risk index by ROC curve analysis. Around 57.8% of the subjects had AC >2.44 and were at the highest risk for developing CAD. This study emphasizes the importance of atherogenic indices in the primary prevention and management of CAD, a growing non-communicable disease.

## Introduction

In our fast-paced modern era, where stress is ubiquitous, the prevalence of dyslipidemia and the burden of coronary artery disease (CAD) are on the rise [1]. CAD, the most common cardiovascular condition, poses a serious risk to people in both developed and developing countries. It has been recognized as one of the leading causes of death globally, placing a significant strain on public health worldwide. Atherosclerosis, the main cause of CAD, is impacted by a number of variables such as oxidative stress, inflammation, and endothelial dysfunction [2]. The lipid profile, also referred to as the coronary risk panel, can be used as a screening technique for determining the risk of CAD [3]. A 2021 World Heart Federation report states that increased low-density lipoprotein cholesterol (LDL-C) was one of the contributing factors to 3.8 million deaths from cardiovascular disease (CVD) [4]. Nearly a quarter (24.8%) of all fatalities in India are attributed to CVD, according to age-standardized estimates from the Global Burden of Disease Research [5]. Both the disease burden and death rate from CAD are rising in India and many other emerging nations [6]. The two main risk factors for atherosclerosis are high levels of total cholesterol (TC) and high levels of LDL-C [7]. Additionally, an increase in triglyceride (TG) and a decrease in high-density lipoprotein (HDL) also contribute to atherosclerosis [8]. A disorder of lipoprotein metabolism characterized by elevated levels of TC, LDL-C, or TGs, low HDL-C, or a combination of these findings is dyslipidemia [9]. The important risk factors contributing to the CAD epidemic are lifestyle and metabolic factors. Most of these are non-modifiable factors; however, dyslipidemia is modifiable and its assessment is quite feasible [10].

Atherogenic indices are feasible to perform as they are mathematical calculations derived from the lipid panel and don't require any additional resources. These indices offer greater predictive capacity for CAD risk than individual lipid parameters, as they integrate multiple lipid measurements to provide a more comprehensive assessment of CAD risk [11]. Castelli's risk index-I (CRI-I), atherogenic coefficient (AC), atherogenic index of plasma (AIP), and Castelli's risk index-II (CRI-II) are various atherogenic indices [12]. AC, a valuable atherogenic index, depends on the importance of HDL-C in determining the risk of CAD [13]. Atherosclerosis and CAD are associated with elevated levels of non-HDL-C [14]. Among the lipid panel measurements, non-HDL-C was found to be the most accurate predictor of both CAD occurrences and strokes [15]. It is well recognized that Asian Indians have a distinct dyslipidemia pattern characterized by higher TG levels and lower HDL-C [1]. Dyslipidemia is a complex condition influenced by various factors such as genetics, lifestyle, diet, and environment. It's fascinating how the burden of dyslipidemia can vary from one region to another. Factors like dietary habits, cultural practices, and genetic diversity likely contribute to this variation. Understanding the pattern of dyslipidemia in the Telangana region is crucial, as previous studies have not sufficiently addressed this. This knowledge is vital for developing effective, population-specific preventive strategies.

Due to the current scenario of increased CAD prevalence and the established role of dyslipidemia as a risk factor for CAD, this study aimed at identifying the prevalent lipid abnormalities in a tertiary care hospital in Telangana. By investigating dyslipidemia in our region, this study can contribute valuable insights that can guide healthcare policies and improve patient outcomes. Furthermore, we aimed at studying the predictive value of atherogenic indices for assessing CAD risk.

## Materials and methods

Study design

This is a retrospective cross-sectional study.

Study area

The study was conducted at the Department of Biochemistry of All India Institute of Medical Sciences, Bibinagar, in Hyderabad, India.

Inclusion criteria

Participants aged 18-80 years of both genders from the outpatient department (OPD) were included. Patients hailing from the surrounding districts such as Yadadri, Medchal, Medak, Rangareddy, and Nalgonda visit our hospital OPDs.

Exclusion criteria

Participants who were ill and admitted to the inpatient ward/ICUs were excluded.

Sample size

The minimum sample size calculated on OpenEpi (Dean AG, Sullivan KM, Soe MM. OpenEpi: Open Source Epidemiologic Statistics for Public Health, Version. www.OpenEpi.com, updated 2013/04/06) taking a prevalence of dyslipidemia as 81.2% [16], α-error as 0.01, and ꞵ-error as 0.05 was 474. Five hundred subjects were included in the study without any age or gender stratification.

Methods

Retrospective data from January 2021 to December 2021 was retrieved from the hospital lab records and analyzed after receiving institutional ethical clearance from the Ethics Committee of All India Institute of Medical Sciences, Bibinagar (approval number: AIIMS/BBN/IEC/JAN/2024/398). Fasting blood samples were collected. Calibration was done at timed intervals, and internal quality control measures were taken adequately as per standard guidelines. An external quality assurance scheme was also performed every month to ensure the comparability of the results with our peer labs. Lipid parameters such as serum TC and HDL were measured by using the enzymatic colorimetric method CHOD-PAP, and serum TGs were measured by the GPO-PAP method in Erba Mannheim Transasia XL 1000 (Transasia Bio-Medicals, Mumbai, India). Serum LDL-C was calculated by using Friedwald's formula: LDL-C=TC-(HDL-C+TG/5) [17].

The National Cholesterol Education Programme Adult Treatment Panel III (NCEP ATP III) Guidelines for the grading of lipid profiles was used [18]. Dyslipidemia was categorized based on Fredrickson's classification [19]. It states that type 1 is elevated chylomicrons, leading to very high TG levels. Type 2A is elevated LDL-C only (without elevated TGs), leading to increased TC. Type 2B is elevated LDL-C and very-low-density lipoprotein (VLDL) (TGs), causing high TC and TGs. Type 3 is elevated intermediate-density lipoprotein (IDL), leading to elevated cholesterol and TGs. Type 4 is elevated VLDL, leading primarily to high TGs with normal or slightly elevated cholesterol. Type 5 is elevated VLDL and chylomicrons, leading to very high TGs and moderately high cholesterol.

Definitions

Dyslipidemia is defined as a disorder of lipoprotein metabolism characterized by elevated plasma levels of total cholesterol, LDL cholesterol, or TGs, low plasma levels of HDL cholesterol, or a combination of these findings [9]. Atherogenic Indices are defined as the ratios of atherogenic dyslipidemia [12]. The Atherogenic Coefficient (AC) is the ratio of Non-High-Density Lipoprotein cholesterol (Non-HDL-C) to HDL-C [13]. CRI-1 and CRI-2 are ratios of TC/HDL-C and LDL-C/HDL-C respectively. AIP implies a logarithm of TG/HDL-C. The atherogenic effects of LDL-C, TG, and VLDL-C in blood, representing all atherogenic Apo lipoprotein-B carrying lipoproteins, can be assessed using non-HDL-C [15]. It is called a ‘poor man’s apolipoprotein B molecule’ [20]. Non-HDL-C is the best predictor of long-term risk of coronary events [21]. Atherogenic Indices were used to predict cardiovascular disease risk [2].

Statistical analysis

The data was analyzed statistically using MedCalc (Version 22.021, MedCalc Software Ltd., Ostend, Belgium). Normality was tested using the Shapiro-Wilk test. Continuous variables such as age and lipid panel parameters were represented as mean and standard deviation (SD) or median and interquartile range (IQR) based on their distribution. Student's t-test was done to check if the difference of means between males and females was significant, and the Mann-Whitney U-test was done for non-normally distributed data. Receiver operating characteristic (ROC) curve analysis was done to determine the cut-offs and to know the best index for the risk prediction of coronary events by taking non-HDL-C as the gold standard as indicated by previous studies [15,20]. p<0.05 was considered statistically significant.

## Results

The study included 500 subjects with 293 males (58.6%) and 207 females (41.4%), and the mean age group for the subjects was 52.87±12.64 years. The summary statistics of the study subjects are tabulated in Table 1. 

**Table 1:** Summary statistics of the study population TC: total cholesterol; TG: triglyceride; HDL-C: high-density lipoprotein cholesterol; LDL-C: low-density lipoprotein cholesterol; VLDL: very-low-density lipoprotein; CRI-1: coronary risk index-1; CRI-2: coronary risk index-2; AIP: atherogenic index of plasma; AC: atherogenic coefficient; non-HDL-C: non-HDL cholesterol; *: mean (SD); #: median (IQR); mg/dl: milligram/deciliter

Variable	Total (n=500)	Males (n=293)	Females (n=207)
Age (years)	52.87 (12.64)^*^	54 (45-65)^#^	52 (43.5-60)^#^
TC (mg/dl)	185.5 (158.3-213.3)^#^	180.7 (43.1)^*^	192.67 (42.59)^*^
TG (mg/dl)	145 (109.3-212.6)^#^	150.8 (108.75-221)^#^	140 (114.12-192.75)^#^
HDL (mg/dl)	51 (44-59.49)^#^	49 (42.92-56)^#^	54 (47.05-62.6)^#^
LDL (mg/dl)	99.36 (35.57)^*^	94.97 (36.38)^*^	105.66 (33.36)^*^
VLDL (mg/dl)	29 (22-42.69)^#^	30.7 (22-42.26)^#^	28 (22.32-38.75)^#^
CRI -1	3.6 (3.01-4.21)^#^	3.69 (3.05-4.24)^#^	3.46 (3.06-4.16)^#^
CRI-2	1.95 (0.68)^*^	1.93 (0.72)^*^	1.96 (0.61)^*^
AIP	0.04 (0.04-0.05)^#^	0.05 (0.04-0.05)^#^	0.04 (0.03-0.05)^#^
AC	2.61 (2.06-3.22)^#^	2.69 (2.06-3.24)^#^	2.46 (2.06-3.15)^#^
Non-HDL-C (mg/dl)	133.35 (110-159.8)^#^	130.08 (2.31)^*^	136.5 (113.56-161.25)^#^

Lipid profile

Serum cholesterol was in the desirable range of 62.8% (n=314) for the total study group. Of this, 62.74% (n=197) were males and 37.26% (n=117) were females. A large proportion of females have higher cholesterol values (p<0.05). Serum TG was above the upper limit for 47% (n= 235) of total subjects. It included 62.97% (n=148) of males and 37% (n=87) of females. Around 23.6% of individuals (n=118) had low HDL values. Females with low HDL (61.02%, n=72) were in a larger proportion compared to males (38.98%, n=46), and this difference was highly significant (p<0.0001) (Table 2).

**Table 2:** Grading of lipid profile according to the NCEP ATP III Guidelines Proportions represented are column percentages. NCEP ATP III: National Cholesterol Education Programme Adult Treatment Panel III; TC: total cholesterol; TG: triglyceride; HDL: high-density lipoprotein cholesterol; LDL-C: low-density lipoprotein cholesterol; VLDL: very-low-density lipoprotein; M: males; F: females; *: p<0.05; **: p<0.01; ***: p<0.0001; mg/dl: milligram/deciliter

Parameter	Grading		Reference range	Total (n=500)		Males (n=293)		Females (n=207)	
(mg/dl)			(mg/dl)	Mean±SD	n (%)	Mean±SD	n (%)	Mean±SD	n (%)
TC		Desirable	<200	160.32±27.92	314 (62.8)	156.79±28.78	197 (67.2)**	166.25±25.42	117 (56.52)**
		Borderline	200-239	216.83±10.51	142 (28.4)	218.24±10.75	76 (25.94)	215.20±10.06	66 (31.88)
		High	≥240	265.86±24.43	44 (8.8)	264.4±31.78	20 (6.83)	267.07±25.21	24 (11.59)
TG		Normal	<150	110.33±24.46	265 (53)	107.11±25.40	145 (49.49)*	114.23±22.77	120 (57.97)*
		High	≥150	242.24±96.38	235 (47)	240.67±77.59	148 (50.51)	244.92±122.36	87 (42.03)
HDL-C		Low	M: <40	39.34±6.66	118 (23.6)	33.91±4	46 (14.6)***	42.81±5.12	72 (34.78)***
			F: <50						
		High	≥60	67.82±7.67	119 (23.8)	68.55±9.04	50 (17.06)	67.29±6.53	69 (33.33)
LDL-C		Optimal	<100	71.66±20.51	242 (48.4)	68.89±21.01	156 (53.24)**	76.08±19.39	86 (41.55)**
		Near-optimal	100-129	113.73±8.77	169 (33.8)	114.13±8.37	93 (31.74)	113.23±9.26	76 (36.71)
		Borderline	130-159	140.57±8.10	65 (13)	141.47±7.82	33 (11.26)	139.58±8.53	32 (15.46)
		High	160-189	169.36±8.42	17 (3.4)	169.30±9.02	8 (2.73)	169.42±8.41	9 (4.35)
		Very high	≥190	204.72±14.02	7 (1.4)	212.56±17.92	3 (1.02)	198.84±8.48	4 (1.93)
VLDL		Normal	≤30	22.17±4.95	267 (53.4)	21.55±5.14	147 (50.17)*	22.89±4.63	123 (59.42)*
		High	>30	48.11±17.08	233 (46.6)	48.32±15.55	146 (49.83)	48.83±21.93	84 (40.58)

Around 12.6% (n=63) of the subjects had deranged LDL alone with no other abnormality.

Dyslipidemia

The burden of dyslipidemia according to Fredrickson's classification in the study population is depicted in Table 3.

**Table 3:** Burden of dyslipidemia according to Fredrickson's classification Proportions represented are column percentages. TC: total cholesterol; TG: triglyceride; HDL-C: high-density lipoprotein cholesterol; LDL-C: low-density lipoprotein cholesterol; VLDL: very-low-density lipoprotein; mg/dl: milligram/deciliter

Category	Description	Total (n=500)	Male (n=293)	Female (n=207)
		n (%)	n (%)	n (%)
Normal	TC <200 mg/dl	139 (27.8)	85 (29.01)	54 (26.08)
	TG <150 mg/dl			
	HDL >50 mg/dl			
	LDL <100 mg/dl			
	VLDL ≤30 mg/dl			
Type 1	Hyperchylomicronemia	90 (18)	64 (21.84)	26 (12.56)
Type 2A	Familial hypercholesterolemia	125 (25)	60 (20.47)	65 (31.4)
Type 2B	Familial combined hyperlipidemia	214 (42.8)	124 (42.32)	91 (43.96)
Type 3	Dysbetalipoproteinemia	121 (24.2)	71(24.23)	50 (24.15)
Type 4	Familial hypertriglyceridemia	90 (18)	64 (21.84)	26 (12.56)
Type 5	Hypertriglyceridemia secondary to other causes	90 (18)	64 (21.84)	26 (12.56)

The overall burden of dyslipidemia was 72.2% (n=361) with 57.6 % (n=208) in males and 42.4% (n=153) in females. The most common dyslipidemia found was familial combined hyperlipidemia in 42.8% (n=214) of individuals. The second most common pattern of dyslipidemia was primary familial hypercholesterolemia which was observed in 25% (n=125) of subjects. Dysbetalipoproteinemia was seen in 24.2% (n=121) of the study subjects.

Atherogenic indices

Around 30.4% (n=152) of the subjects had non-HDL between 130 and 160 mg/dl and 24% (n=120) had >160 mg/dl of non-HDL-C. Around 39.8% (n=199), 31.2% (n=156), and 29% (n=145) of the subjects had non-HDL-C <125 mg/dl, 125-155 mg/dl, and >155 mg/dl, respectively. The ROC curve analysis done for determining the cut-offs of various atherogenic risk indices is shown in Table 4.

**Table 4:** ROC curve analysis in total study subjects for establishing the cut-offs for various atherogenic indices for estimating cardiovascular risk ROC: receiver operating characteristic; TC: total cholesterol; TG: triglyceride; HDL-C: high-density lipoprotein cholesterol; LDL-C: low-density lipoprotein cholesterol; VLDL: very-low-density lipoprotein; CRI-1: coronary risk index-1; CRI-2: coronary risk index-2; AIP: atherogenic index of plasma; AC: atherogenic coefficient; AUC: area under the curve; SE: standard error; CI: confidence interval; Z: test statistics; P: significance; mg/dl: milligram/deciliter

Variable	AUC	SE	95% CI	Z	P	Youden index J	Sensitivity	Specificity	Criterion
TC (mg/dl)	0.975	0.00518	0.957-0.987	91.699	<0.0001	0.8175	92.28	89.47	>180
TG (mg/dl)	0.729	0.0224	0.687-0.767	10.214	<0.001	0.3633	61.76	74.56	>152.1
HDL (mg/dl)	0.589	0.0258	0.544-0.632	3.428	0.001	0.1719	76.84	40.35	>45.87
LDL (mg/dl)	0.933	0.0109	0.907-0.953	39.672	<0.0001	0.7474	80.88	93.86	>102.77
VLDL (mg/dl)	0.732	0.0222	0.691-0.771	10.452	<0.0001	0.3676	61.76	75	>30.42
CRI -1	0.845	0.0174	0.811-0.876	19.819	<0.001	0.5523	81.99	73.25	>3.44
CRI-2	0.851	0.0168	0.817-0.881	20.829	<0.001	0.5597	71.32	84.65	>2
AIP	0.504	0.0253	0.460-0.549	0.169	0.866	0.03193	83.46	19.74	≤0.05
AC	0.852	0.017	0.818-0.882	20.736	<0.001	0.5597	82.72	73.25	>2.44

Using the new cut-offs established with ROC curve analysis, the study population was classified as tabulated in Table 5.

**Table 5:** Grading of lipid profile according to the new cut-offs obtained from the ROC curve analysis Proportions represented are column percentages. ROC: receiver operating characteristic; TC: total cholesterol; TG: triglyceride; HDL-C: high-density lipoprotein cholesterol; LDL-C: low-density lipoprotein cholesterol; VLDL: very-low-density lipoprotein; mg/dl: milligram/deciliter; M: males; F: females

Parameter	Cut-offs	Total (n=500)	Male (n=293)	Female (n=207)
(mg/dl)	(mg/dl)	n (%)	n (%)	n (%)
TC	>180	275 (55)	142 (48.46)	133 (64.25)
TG	>152.1	226 (45.2)	145 (49.49)	81 (39.13)
HDL-C	M: <44.6	122 (24.4)	98 (33.44)	24 (11.59)
	F: <40.2			
LDL-C	>102.77	234 (46.8)	126 (43)	108 (52.17)
VLDL	>30.42	225 (45)	145 (49.49)	80 (38.65)

The best predictor for CAD is AC with a cut-off value of >2.44, a sensitivity of 82.72%, a specificity of 73.25%, and an AUC of 0.852. Around 42.2% (n=211) of the study population were found to be normal, among which 54.03% (n=114) were males and 45.97% (n=97) were females. Around 57.8% (n=289) of the total were found to be at increased risk for developing CAD, among which 61.9% (n=179) were males and 38.06% (n=110) were females.

## Discussion

One of the prime causes of the increased morbidity and mortality in our society is CAD. Dyslipidemia may affect the coronary vasculature leading to atherosclerosis followed by ischemia. The most crucial step in the primary prevention of CAD is to identify individuals with dyslipidemia and treat them appropriately, thus halting the progression to atherosclerosis and CAD. Atherogenic indices play a major role in predicting the risk of CAD. In lieu of this scenario, our study estimated the burden of dyslipidemia and predicted the risk of CAD through atherogenic indices among the patients coming to a tertiary care hospital in Telangana. The overall burden of dyslipidemia in our study was 72.2% with familial combined hyperlipidemia in 42.8% (n=214) of individuals. AC was found to be the most sensitive and specific cardiovascular risk index by ROC curve analysis. Around 57.8% of the subjects had AC >2.44 and were at the highest risk for developing CAD.

High TG levels have been shown in the past to be an indicator of CAD risk [14,22]. TG contributes to the pathogenesis of CAD through processes, such as its action on the metabolism of other lipoproteins, transport proteins, enzymes, coagulation, and endothelial dysfunction. Diet, age, lifestyle, medication therapy, and metabolic diseases are some of the factors that affect TG levels [22]. Hypertriglyceridemia was observed in 47% (n=235) in our study. Out of which, 62.97% (n=148) were male subjects and 37.02% (n=87) were female subjects. The gender variation seen in the present study is in concordance with the Indian Council of Medical Research-India Diabetes (ICMR-INDIAB) study [16].

In the ICMR-INDIAB-17 study, the prevalence of hypertriglyceridemia in the nation was found to be 32.1%; however, the burden in the Telangana population was 21-23% [16]. In our study, we observed a higher proportion of hypertriglyceridemia. This finding may be attributed to factors such as limited awareness of healthy lifestyle practices and the consumption of energy-dense foods. Notably, the low socioeconomic status of the population could contribute to these patterns. Unfortunately, due to the retrospective study design, we lacked specific data on diet and lifestyle. Western populations, South Asians, and Indians have higher TG than high TC in comparison with the UK populations and Europeans [23,24].

There were limited studies conducted in India on cholesterol and other lipoprotein lipids in large samples for the past 30 years [23]. We found that the proportion of subjects with hypercholesterolemia in our study was 37.2% (n=186). It is higher than the reported prevalence rates of 18-25% in Telangana according to the ICMR-INDIAB study [16]. The higher burden noticed in our study might be due to the mixture of rural and urban populations in our clinical setting. Contrary to the Western population in high-income countries like North America and Western Europe where the mean cholesterol concentration was low, we found a higher burden of hypercholesterolemia which could be attributed to the consumption of highly processed foods, low socioeconomic status, and sedentary lifestyle [25]. Around 12.6% (n=63) of the subjects had deranged LDL alone with no other abnormality. LDL >130 mg/dl was found in 17.8% (n=89) of study subjects with 49.44% (n=44) of males and 50.56% (n=45) of females comparable to the data of Telangana in the ICMR-INDIAB study [16]. HDL is considered to be a "protective lipid factor" [23,26]. Around 23.6% (n=118) of the study subjects had low HDL. Discordant with the ICMR-INDIAB study, we observed that the majority of the subjects with low HDL (61.02%, n=72) were females as compared to males (38.98%, n=46) [16]. The observed gender variation could be influenced by hormonal factors and the distinct lifestyle choices made by each gender.

In our study, an ROC curve analysis (Table 4) showed that among the lipid profile parameters, TC is a standalone risk predictor with a 92.28% sensitivity and an 89.47% specificity. The Youden index that assesses the balance between sensitivity and specificity of a test was 0.82. AC is the best atherogenic index with a sensitivity of 82.72% and a specificity of 73.25%, and the Youden index was found to be 0.56 which is in concordance with previous studies [2,27]. Furthermore, AC is the single best atherogenic index as it is calculated taking the ratio of non-HDL-C that accounts for the atherogenicity of all the lipoproteins and HDL-C which is the single protective lipid factor. The proportion of individuals with non-HDL-C more than 160 mg/dl was 24% (n=120), and ≥130 mg/dl was about 54.4% (n=272) which agrees with Guptha et al. [28].

Our study acknowledges its relatively small sample size and retrospective nature. Unfortunately, due to the retrospective design, we lacked specific data on diet and medication use. These limitations may have influenced our findings, and it's essential to interpret them with caution. Telangana's population faces distinct health challenges influenced by genetics, lifestyle, and cultural factors. By examining dyslipidemia, our research provides insights directly catering to the well-being of this population. To better understand the burden of dyslipidemia and the risk of CAD in this population in a better way, large-scale cohort studies focusing on diet and socio-demographic factors could be conducted in this region with a focus on diet patterns, socio-demographic factors, and other local determinants of health. Additionally, randomized controlled trials can evaluate interventions aimed at effectively reducing dyslipidemia in Telangana.

## Conclusions

The overall burden of dyslipidemia was 72.2%. The most common dyslipidemia found was familial combined hyperlipidemia in 42.8% (n=214) of individuals. AC was found to be the most sensitive and specific cardiovascular risk index by ROC curve analysis. Around 57.8% of the subjects had AC >2.44 and were at the highest risk for developing CAD. The incidence of CAD is increasing tremendously; thus, there is an urgent need for screening high-risk individuals to facilitate the primary prevention of CAD as it is well-known that "an ounce of prevention is better than a pound of cure." This study also reinforces the need for community education programs and public health initiatives focusing on dyslipidemia. 

## References

[1] Joshi SR, Anjana RM, Deepa M (2014). Prevalence of dyslipidemia in urban and rural India: the ICMR-INDIAB study. PLoS One.

[2] Li Y, Feng Y, Li S, Ma Y, Lin J, Wan J, Zhao M (2023). The atherogenic index of plasma (AIP) is a predictor for the severity of coronary artery disease. Front Cardiovasc Med.

[3] França CN, Mendes CC, Ferreira CE (2018). Time collection and storage conditions of lipid profile. Braz J Med Biol Res.

[4] Murray CJ (2022). The Global Burden of Disease Study at 30 years. Nat Med.

[5] Pirillo A, Casula M, Olmastroni E, Norata GD, Catapano AL (2021). Global epidemiology of dyslipidaemias. Nat Rev Cardiol.

[6] Gupta R, Wood DA (2019). Primary prevention of ischaemic heart disease: populations, individuals, and health professionals. Lancet.

[7] Sharma S, Gaur K, Gupta R (2024). Trends in epidemiology of dyslipidemias in India. Indian Heart J.

[8] Cai G, Shi G, Xue S, Lu W (2017). The atherogenic index of plasma is a strong and independent predictor for coronary artery disease in the Chinese Han population. Medicine (Baltimore).

[9] Misra S, Lyngdoh T, Mulchandani R (2022). Guidelines for dyslipidemia management in India: a review of the current scenario and gaps in research. Indian Heart J.

[10] Teo K, Chow CK, Vaz M, Rangarajan S, Yusuf S (2009). The Prospective Urban Rural Epidemiology (PURE) study: examining the impact of societal influences on chronic noncommunicable diseases in low-, middle-, and high-income countries. Am Heart J.

[11] Dharmaraj S, Rajaragupathy S, Denishya S (2022). A descriptive study of atherogenic indices in patients admitted to a tertiary care hospital. Cureus.

[12] Tien YT, Wang LJ, Lee Y, Lin PY, Hung CF, Chong MY, Huang YC (2023). Comparative predictive efficacy of atherogenic indices on metabolic syndrome in patients with schizophrenia. Schizophr Res.

[13] Gol RM, Rafraf M, Jafarabadi MA (2021). Assessment of atherogenic indices and lipid ratios in the apparently healthy women aged 30-55 years. Arterial Hypertens.

[14] Ference BA, Ginsberg HN, Graham I (2017). Low-density lipoproteins cause atherosclerotic cardiovascular disease. 1. Evidence from genetic, epidemiologic, and clinical studies. A consensus statement from the European Atherosclerosis Society Consensus Panel. Eur Heart J.

[15] Carr SS, Hooper AJ, Sullivan DR, Burnett JR (2019). Non-HDL-cholesterol and apolipoprotein B compared with LDL-cholesterol in atherosclerotic cardiovascular disease risk assessment. Pathology.

[16] Anjana RM, Unnikrishnan R, Deepa M (2023). Metabolic non-communicable disease health report of India: the ICMR-INDIAB national cross-sectional study (ICMR-INDIAB-17). Lancet Diabetes Endocrinol.

[17] Friedewald WT, Levy RI, Fredrickson DS (1972). Estimation of the concentration of low-density lipoprotein cholesterol in plasma, without use of the preparative ultracentrifuge. Clin Chem.

[18] (2001). Executive summary of the third report of the National Cholesterol Education Program (NCEP) expert panel on detection, evaluation, and treatment of high blood cholesterol in adults (adult treatment panel III). JAMA.

[19] Mosca S, Araújo G, Costa V (2022). Dyslipidemia diagnosis and treatment: risk stratification in children and adolescents. J Nutr Metab.

[20] Sniderman A, Langlois M, Cobbaert C (2021). Update on apolipoprotein B. Curr Opin Lipidol.

[21] Brunner FJ, Waldeyer C, Ojeda F (2019). Application of non-HDL cholesterol for population-based cardiovascular risk stratification: results from the Multinational Cardiovascular Risk Consortium. Lancet.

[22] Laufs U, Parhofer KG, Ginsberg HN, Hegele RA (2020). Clinical review on triglycerides. Eur Heart J.

[23] Gupta R, Rao RS, Misra A, Sharma SK (2017). Recent trends in epidemiology of dyslipidemias in India. Indian Heart J.

[24] Volgman AS, Palaniappan LS, Aggarwal NT (2018). Atherosclerotic cardiovascular disease in South Asians in the United States: epidemiology, risk factors, and treatments: a scientific statement from the American Heart Association. Circulation.

[25] Farzadfar F, Finucane MM, Danaei G (2011). National, regional, and global trends in serum total cholesterol since 1980: systematic analysis of health examination surveys and epidemiological studies with 321 country-years and 3·0 million participants. Lancet.

[26] Xiang AS, Kingwell BA (2019). Rethinking good cholesterol: a clinicians' guide to understanding HDL. Lancet Diabetes Endocrinol.

[27] Niroumand S, Khajedaluee M, Khadem-Rezaiyan M (2015). Atherogenic index of plasma (AIP): a marker of cardiovascular disease. Med J Islam Repub Iran.

[28] Guptha S, Gupta R, Deedwania P (2014). Cholesterol lipoproteins and prevalence of dyslipidemias in urban Asian Indians: a cross sectional study. Indian Heart J.

